# 560. Evaluation of Optimal Methylprednisolone Dose in Patients with Covid 19

**DOI:** 10.1093/ofid/ofab466.758

**Published:** 2021-12-04

**Authors:** Jose Luis Lamas Ferreiro, Judith Álvarez Otero, Fernando Maroto Piñeiro, Iolanda Abalde Ortega, Marta Rodríguez Villar, Sonia Morón Losada, Irea Vidal González, Alejandra Canoa Rico, Laura Fernández González, Emilia Fernández Fernández, Marta Costas Vila, Ana Sanjurjo Rivo, Francisco Fernández Fernández, Jose Manuel Paz Ferrín, Alexandra Arca Blanco, María Alonso, Javier De la Fuente

**Affiliations:** 1 Ribera Povisa Hospital, Vigo, Galicia, Spain; 2 Hospital Povisa, Vigo, Galicia, Spain

## Abstract

**Background:**

Optimal dose of methylprednisolone in patients with moderate or severe COVID-19 is unclear. In our hospital, the use of 250-500 mg/day of methylprednisolone was frequent in the first wave of the pandemic. Lower dose were recommended in our protocol since September 2020. The aim was to evaluate the impact of methylprednisolone dose in the outcome of patients with moderate or severe COVID-19.

**Methods:**

This is a retrospective and observational study. Inclusion criteria: SARS-CoV-2 infection diagnosed by PCR, admission to our hospital between March 2020 and February 2021, SatO2 < 94% or SatO2/FiO2 < 447. Two treatment groups were compared: patients treated with 0.5-1.5 mg/kg/day (group 1) and patients treated with more than 1.5 mg/kg/day (group 2). The primary outcome analyzed was orotracheal intubation (OTI) or death from any cause at 28 days after admission. Differences in demographic, clinical and laboratory characteristics between treatment groups were analyzed. Variables with P < 0.1 were included in a binary logistic regression model, calculating a propensity score for assigning each patient to group 1 treatment. Bivariate analysis was performed to identify variables associated with worst outcome. Finally, Cox regression was performed including treatment group, propensity score as covariate and all the variables with P< 0.05 in the bivariate analysis.

**Results:**

285 patients were included, 197 in group 1 and 88 in group 2. The median age was 73 years, 52,3% were male. Mortality or OTI at 28 days was 24,9%. There was a higher proportion of patients in group 1 with COPD (9,6% vs 1.1%, P< 0.01), dyspnea (60.4% vs 45.5%, P=0.01), sepsis (22.8% vs 13.6%, P=0.07). Patients in group 2 had more impaired consciousness (18.2% vs 8.6%, P=0.02). The median of lymphocytes count was lower in group 1 (900 vs 1025, P=0.01). There were no differences in the primary outcome between treatment groups (26.1% in the group 2 vs 24.4% in the group 1, P=0.7).

**Conclusion:**

The use of high dose of methylprednisolone compared with intermediate dose is not associated with a better outcome in patients with moderate or severe COVID-19.

**Disclosures:**

**All Authors**: No reported disclosures

